# Challenges of the next decade for the Asia Pacific region: 2010 International Conference in Bioinformatics (InCoB 2010)

**DOI:** 10.1186/1471-2164-11-S4-S1

**Published:** 2010-12-02

**Authors:** Shoba Ranganathan, Christian Schönbach, Kenta Nakai, Tin Wee Tan

**Affiliations:** 1 Department of Chemistry and Biomolecular Sciences and ARC Centre of Excellence in Bioinformatics, Macquarie University, Sydney NSW 2109, Australia; 2 Department of Biochemistry, Yong Loo Lin School of Medicine, National University of Singapore, 8 Medical Drive, Singapore 117597; 3 Department of Bioscience and Bioinformatics, Kyushu Institute of Technology, Fukuoka 820-8502, Japan; 4 Laboratory of Functional Analysis in silico, Human Genome Center , The Institute of Medical Science , The University of Tokyo, Tokyo 108-8639, Japan

## Abstract

The 2010 annual conference of the Asia Pacific Bioinformatics Network (APBioNet), Asia’s oldest bioinformatics organisation formed in 1998, was organized as the 9^th^ International Conference on Bioinformatics (InCoB), Sept. 26-28, 2010 in Tokyo, Japan. Initially, APBioNet created InCoB as forum to foster bioinformatics in the Asia Pacific region. Given the growing importance of interdisciplinary research, InCoB2010 included topics targeting scientists in the fields of genomic medicine, immunology and chemoinformatics, supporting translational research. Peer-reviewed manuscripts that were accepted for publication in this supplement, represent key areas of research interests that have emerged in our region. We also highlight some of the current challenges bioinformatics is facing in the Asia Pacific region and conclude our report with the announcement of APBioNet’s 100 BioDatabases (BioDB100) initiative. BioDB100 will comply with the database criteria set out earlier in our proposal for Minimum Information about a Bioinformatics and Investigation (MIABi), setting the standards for biocuration and bioinformatics research, on which we will report at the next InCoB, Nov. 27 – Dec. 2, 2011 at Kuala Lumpur, Malaysia.

## Background

Since 1998, the Asia Pacific Bioinformatics Network (APBioNet) [1] has strived to bring together scientists from diverse disciplines working together to advance the frontiers of bioinformatics in the region. In 2002, the bioinformatics research effort was sufficiently well established to encourage scientists from Thailand and the APBioNet to launch InCoB2002 (the International Conference on Bioinformatics, 2002) in Bangkok and adopt this meeting as their annual conference. Subsequent InCoB meetings were hosted in strategic venues in the region, particularly to stimulate greater awareness and urgency amongst local biologists for the need to transform their research capability by including tools and techniques in bioinformatics and computational biology.

### Promoting bioinformatics development through conferences

Over the past eight years of InCoB conferences (Bangkok, Thailand (2002); Penang, Malaysia (2003); Auckland, New Zealand (2004); Busan, South Korea (2005); New Delhi, India (2006) [2]; Hong Kong (2007) [3]; Taipei, Taiwan (2008) [4] and Singapore (2009) [5,6]) and associated satellite workshops and meetings, we have seen a healthy growth in the development and advancement of bioinformatics, commensurate with an increase in the number of scientists who moved into bioinformatics from other fields and the number of students who have taken an interest in bioinformatics. Consequently, there has been a significant growth in the number of publications in bioinformatics, as well in "wetlab" biology, containing computational techniques. Last but not least, the quality of publications and the standard of research reported in papers originating from China, India and the Southeast Asian region have rapidly improved [personal communication from Elsevier and others].

Many Asian-trained students have moved on farther afield and have found research laboratories in North America or Europe to advance their careers during post-doctoral research, or seek positions as principal investigators and academic faculty. Due to a robust groundswell of interest, there is no brain-drain. On the contrary a "brain-overflow" appears to emerge, and looking ahead, there is no doubt that the region's top universities and research institutions will continue to be an active trainer of bioinformatics researchers for the rest of the world and a breeding ground for promising bioinformatics students. Many new conferences, meetings and journals in bioinformatics and computational biology which have emerged and sustained themselves in the region are one result of the growing strength of bioinformatics and demand in research dissemination.

### Promoting widespread awareness of bioinformatics need

Factors driving this rapid growth include a strong awareness that the curriculum of a typical undergraduate life science course has to include a significant level of training in computer science and computational biology, supported by the ingress of high quality researchers from computer science and ICT. Where skillsets are lacking, many universities in our region have also incorporated conversion courses for biologists wishing to acquire computational skills through post-graduate education, such as the popular Bioinformatics Masters by coursework degree programs, particularly in India. In this regard, over the past decade, APBioNet has held numerous training activities in partnership with organisations such as the S* Life Science Informatics Alliance, the Federation of Asian-Oceanic Biochemists and Molecular Biologists (FAOBMB), Asia-Pacific International Society for Molecular Biologists (A-IMBN), the Institute Pasteur, the EUAsiaGrid initiative, inter-governmental agencies such as ASEAN and APEC, and others. These efforts have built a base of bioinformatics competency and stimulated other initiatives in training and education such as the Thai hypercourse in bioinformatics, the ASEAN Virtual Institute of Science and Technology (AVIST) and the Japan-based Asian Bioinformatics Research and Education Network (ABREN, [7]). Overall, these efforts have contributed to the growth of skilled bioinformaticians over the past decade.

To maintain the interconnectedness of our regional bioinformaticians with the rest of the world, APBioNet was invited to be a regional affiliate of the International Society for Computational Biology (ISCB) [8] by P.E. Bourne, the ISCB President in 2002. The presence and support of succeeding Presidents, Board Directors and senior ISCB members, notably M. Gribskov (InCoB 2006), M. Waterman (InCoB 2006), B. Rost (InCoB 2007), T. Gaasterland (InCoB 2008), and C. Sander (InCoB 2009), had significant impact on the growth and prestige of the field in the Asia Pacific region.

### Supporting a culture of inclusivity

During the past ten years, national Bioinformatics societies have also formed in our region, as well as discipline specific groups led by thought leaders in Asia Pacific. In this regard, the APBioNet has through InCoB conference and other workshops, maintained strong ties of collaboration continually and lent support for national initiatives wherever possible. For instance, in the current InCoB2010 conference, partnership with the Japanese Society for Bioinformatics (JSBi) [9], the Chem-Bio Informatics Society of Japan (CBI) [10] and the International Immunomics Society (IIMMS) [11] lent it a strong cross fertilization of ideas from the chemoinformatic and the immunoinformatic communities and has helped to sustain the strong inter-disciplinary and inclusive approach of APBioNet.

### Encouraging bioinformatics research output

To increase the submission of research papers with potential high impact and citation rates, APBioNet started in 2006 to publish the best submitted InCoB papers in a dedicated BMC Bioinformatics supplement [2]. By 2009, manuscripts from APBioNet members diversified and increased in quality and sophistication with computational biology articles published in BMC Genomics [5] as well as BMC Bioinformatics [6], while this year, due to overwhelming support, we have three journal supplements, continuing with BMC Genomics, BMC Bioinformatics [12] and adding on Immunome Research [13]. This supplement represents the majority of accepted papers, spanning sequence, genome and transcriptome analyses; networks, pathways and systems biology; disease informatics; data and text mining; and structural bioinformatics.

### Rigour in review policy

The manuscripts submitted to InCoB2010 proceedings were, in the main, subject to two rounds of peer-review by at least three reviewers, from the APBioNet/InCoB program committee members and external experts as required (Additional file 1). InCoB2010 provided multi-track submissions, with the inclusion of late breaking abstracts from recent publications and for showcasing technology developments. The aim of the editors was to select the best papers from Asia Pacific countries, with a few from European and North American countries. From the 108 full paper submissions, 50 were shortlisted for oral presentation. This supplement features 25 papers, with another 15 papers in BMC Bioinformatics and six in Immunome Research, reflecting an overall acceptance rate of 42.6%. To encourage early career researchers, a few short articles will appear in the online journals, Bioinformation [14] and IPSJ Transactions on Bioinformatics [15]. A brief review of the various themes in this supplement, as follows, serves to illustrate the breadth and depth of research that is taking place in our region.

#### Sequence, genome and transcriptome analysis

Huang *et al*. [16] present a sequence-based predictor to identify the functional residues in a RNA-binding protein while Le *et al*. [17] have uncovered sequence-dependent histone variant positioning signatures, providing additional insights into epigenetic regulatory mechanisms of many important cellular processes. Lin *et al*. [18] have improved secondary structure prediction using short subsequences with local structural similarity.

NGSQC, a next generation sequence analysis pipeline is presented by Dai *et al*. [19], while Li *et al*. [20] have used next generation sequence data to discover and characterize medaka miRNA genes.

Chen *et al*. [21] present UPS 2.0, an updated version of their software for selecting unique probes for oligonucleotide microarrays for pangenomic and genomic studies, while Ogura *et al*. [22] have applied probe design to the development of an in vitro homology search array.

Rather than cluster homologous sequences alone, Jia *et al*. [23] have clustered orthologous sequences at the transcript level while the analysis of Meng *et al*. [24] present a first global view of RNA editing in plant nuclear transcripts.

#### Networks, pathways and systems biology

Unravelling gene regulatory networks is a topic of intense research. Chen *et al*. [25] have explored dynamic gene regulatory interactions from gene expression data, while Summer and Perkins [26] have formulated a functional data analysis approach to identify nonlinear models of gene regulatory networks. Ayukawa *et al*. [27] present a standardized method to integrate operator sequences to the regulatory region of a plasmid.

Protein interactions are compounded by the problem of a protein being simultaneously present in different subcellular compartments. PathLocdb is a comprehensive database for subcellular localization of metabolic pathway with application for multiple localization analysis [28]. To uncover the modular structure of protein interaction networks, Liu *et al*. [29] propose a new density-based algorithm (ADHOC).

#### Disease informatics

Yang [30]* et al*. have compiled dbDEMC: a database of differentially expressed miRNAs in human cancers, to improve the classification, diagnosis and treatment of human cancers, while Shimokawa *et al*. [31] present iCOD: an integrated clinical omics database based on the systems-pathology view of disease. With the health of aging populations providing a major challenge to the medical profession, Kwon *et al*. [32] present Gerontome: a web-based database and analysis server for aging-related genes.

To assist the development of peptide-based diagnostics, therapeutics and vaccines, Wee *et al*. [33] have developed a novel predictor for B cell epitopes, while Yoo *et al*. [34] address the issue of predicting AIDS disease progression using HIV structural gp120 profiles. At a much wider level, Nagaraj *et al*. [35] present the human hereditary diseasome, to explore relationships among gene attributes and thereby characterise evolutionary trends associated with disease genes.

#### Data and text mining

Laurila *et al*. [36] introduce the first rule-based approach for the extraction of mutation impact on protein properties, using text mining and semantic web technologies. To mine the mutational content of 12 Drosophila genomes in a phylogenetically relevant manner, Yampolsky and Bouznier [37] present a tool for the genome-wide analysis of frequencies and patterns of amino acid substitutions.

#### Structural bioinformatics

Finding novel drugs has been assisted by the availability of protein structures. Grover *et al*. have investigated the anti-cancerous potency of the potential herbal drug, Withaferin, using mammalian 20S proteasomes [38] and propose a novel mechanism of potential action associated with Nuclear Factor kappa B suppression [39]. Clinchiu *et al*. [40] propose TSCC: a Two-Stage Combinative Clustering for virtual screening using protein-ligand interactions and physical-chemical features, to assist drug design.

### Identifying new key challenges and tackling new issues

These research areas show that in the past decade, we in the Asia Pacific have achieved much in bioinformatics research, development and education, either through the APBioNet or in collaboration with others. We have also stimulated others to create their own independent efforts to promote bioinformatics. The early phase of growth, focusing on awareness, education and meetings has been set in motion with good sustainable progress. The publications in this journal supplement and those from previous conferences testify to the spectrum of activities and the quality achieved in a cross section of our research community.

Going forward, several new initiatives led by APBioNet will shape the future of bioinformatics and computational biology in the region. To promote increasing quality of research publications and to sustain this growth in bioinformatics, we have identified several key challenges and problems, not necessarily peculiar to our region, but nonetheless of critical importance to its future growth, which APBioNet can address at the organizational level:

1. The need for a standards-based approach towards more robust software development and software interoperability.

2. The lack of persistence of such databases and the interoperability of data content.

3. The potential lack of *in silico* research reproducibility and the promotion of high quality peer-review process.

4. The growing problem of author ambiguity associated with the nature of transliterated Asian names and the issues of non-repudiability, duplicative "me-too" research and plagiarism.

5. E-science infrastructure of our region not keeping pace with the burgeoning numbers of researchers, both in bioinformatics and in bioinformatics-driven biology, demanding better computing and network performance in other regions, particularly in North America and Europe.

These issues are potential threats to the sustainable growth of bioinformatics in our region, and APBioNet, through InCoB and other meetings, has started to address them through concrete initiatives, novel efforts and synergistic partnership with other organizations.

### APBioNet initiatives to seek collaborators and partners

In InCoB2009, APBioNet launched the MIABi initiative, the Minimum Information about a Bioinformatics Investigation.  In the MIABi framework, work is in progress to crystallize basic key points of agreement on what constitutes a set of minimum information criteria. All authors of this issue of the InCoB2010 conference supplement have agreed to MIABi compliance.

Discussions have been initiated with colleagues in the International Society for Biocuration (ISB) [41] on their initiative for minimum standards in biological database curation to ensure harmonization of standards. The procedural issues for implementing such as system include the following aspects which will be addressed in the paper included in this supplement [42]:

a. Minimum Information about a bioinformatics investigation (MIABi)

b. Data and software persistence and perpetuity

c. Reinstantiability and reproducibility

d. Author and Contributor Identity Disambiguation

e. Standardized Terminology

f. Infrastructural and informational interoperability such as use of international computational grids and cloud computing

### BioDB100, the 100 BioDatabases initiative

To prove that the whole suite of procedures and protocols mentioned above will work on real world live systems, we propose to implement from this year, a BioDB100 challenge: the challenge to build 100 sustainable BioDatabases and datasets to test them against the MIABi standards compliance process and the protocols for re-instantiability, reproducibility, version control and author disambiguation. To incentivise our stakeholders to contribute and participate, we have to offer some solutions for persistence and some workable ideas for interoperability. In this regard, we are currently in discussions with EUAsiaGrid and various Asia Pacific partners to provision for data storage space on a grid or cloud platform or both. Such high performance computational platforms can also lend itself to workflow integration and automation of database maintenance. We plan to request the developers and curators of secondary databases who rely on key public databases to design their database management system to automate the update of their boutique secondary databases based on new entry updates regularly. We hope that they will continue to build their databases in compliance with standards and checklists that emerge from international community initiatives such as those from MIABi [42], the Biocuration conference series [41] and the MIBBI project (Minimum Information for Biological and Biomedical Investigations) [43].

## Conclusion

Considering the scope and depth of topics covered in this issue, Asia Pacific bioinformatics research continues to increase its level of achievement. This area is now a core research discipline in our educational and research institutions, encompassing not only the “-omics” sciences and systems biology, but also in translational research and personalized medicine. Moreover, we are taking steps in the direction of thought leadership and scientific policy which we hope will set the example for a new generation of scientists who fully embrace the new biology of today that is increasingly information and technology-driven, with knowledge generation dependent on applications of physical and computer sciences.

## Competing interests

None declared.

## Supplementary Material

Additional File 1 APBioNet InCoB2010 Program Committee members and reviewers Click here for file

Additional File 2 InCoB2010 sponsors Click here for file
