# Supplementary material for: Challenges of the next decade for the Asia Pacific region: 2010 International Conference in Bioinformatics (InCoB 2010)
Source: BMC Genomics. 2010 Dec 2;11(Suppl 4):S1. doi: 10.1186/1471-2164-11-S4-S1 (PMC3005919; doi:10.1186/1471-2164-11-S4-S1)
Supplement: Additional File 1 — APBioNet InCoB2010 Program Committee members and reviewers [file 1471-2164-11-S4-S1-S1.pdf]

Challenges of the next decade for the Asia Pacific region: 2010 International Conference in Bioinformatics (InCoB 2010)

Shoba Ranganathan, Christian Schönbach, Kenta Nakai and Tin Wee Tan

Additional File 1: APBioNet InCoB2010 Program committee members and reviewers

InCoB Program Committee (in alphabetical order)

1. Tatsuya Akutsu, Kyoto University, Japan
2. Peter Antal, Budapest University of Technology and Economics, Hungary
3. Vladimir Bajic, King Abdullah University of Science and Technology, Saudi Arabia
4. Christopher J. O. Baker, University of New Brunswick, Canada
5. Sergio Baranzini, University of California, San Francisco, USA
6. Alex Bateman, The Wellcome Trust Sanger Institute, UK
7. Jonas Bergman Laurila, University of New Brunswick, Canada
8. Zhiwei Cao, Tongji University, P.R. China
9. Filippo Castiglione, IAC "M. Picone", Italy
10. Alan Christoffels, South African National Bioinformatics Institute, South Africa
11. Adrian Cootes, Macquarie University, Australia
12. Anne D Groot, EpiVax Inc., USA
13. David S. DeLuca, Dana-Farber Cancer Institute, Harvard University, USA
14. Frank Eisenhaber, Bioinformatics Institute, A*STAR, Singapore
15. Darren Flower, Aston University, UK
16. Ge Gao, Center for Bioinformatics, Peking University, P.R. China
17. Takashi Gojobori, National Institute of Genetics, Japan
18. Susumu Goto, Kyoto University, Japan
19. M. Michael Gromiha, Computational Biology Research Center, AIST, Japan
20. Taizo Hanai, Kyushu University, Japan
21. Chia-Lang Hsu, National Yang-Ming University, Taiwan
22. Wen-Lian Hsu, Academia Sinica, Taiwan
23. Chun-Hsi Huang, University of Connecticut, USA
24. Mitsuhiro Itaya, Keio University, Japan
25. Hideo Iwasaki, Waseda University, Japan
26. Javed Mohammed Khan, Macquarie University, Australia
27. Daisuke Kiga, Tokyo Institute of Technology, Japan
28. Akira Kinjyo, Osaka University, Japan
29. Kengo Kinoshita, Tohoku University, Japan
30. Tetsuya Kobayashi, University of Tokyo, Japan
31. Akihiko Konagaya, Tokyo Institute of Technology, Japan
32. Hiroyuki Kurata, Kyushu Institute of Technology, Japan
33. Igor V. Kurochkin, Bioinformatics Institute, A*STAR, Singapore
34. Chih Lee, University of Connecticut, USA
35. Marie-Paule Lefranc, Université Montpellier 2, CNRS, IFR3, France
36. Ole Lund, Technical University of Denmark, Denmark
37. Hiroshi Mamitsuka, Kyoto University, Japan
38. Satoshi Murata, Tohoku University, Japan

39. Hideo Matsuda, Osaka University, Japan
40. Santo Motta, University of Catania, Italy
41. H.A. Nagarajaram, Centre for DNA Fingerprinting and Diagnostics, India
42. Kenta Nakai (PC Co-Chair), University of Tokyo, Japan
43. Haruki Nakamura, Osaka University, Japan
44. See-Kiong Ng, Institute for Infocomm Research, Singapore
45. Jun Ni, The University of Iowa, USA
46. Takenao Ohkawa, Kobe University, Japan
47. Masahiro Okamoto, Kyushu University, Japan
48. Yasushi Okazaki, Saitama Medical University, Japan
49. Ashwini Patil, University of Tokyo, Japan
50. Nikolai Petrovsky, Flinders Medical Centre, Australia
51. Jagath C. Rajapakse, Nanyang Technological University, Singapore
52. Shoba Ranganathan, Macquarie University, Australia
53. Timothy Ravasi, King Abdullah University of Science and Technology, Saudi Arabia
54. Rintaro Saito, Keio University, Japan
55. Yasubumi Sakakibara, Keio University, Japan
56. Christian Schönbach (PC Co-Chair), Kyushu Institute of Technology, Japan
57. Masakaso Sekijima, Tokyo Institute of Technology, Japan
58. Mohammad Tabrez Anwar Shamim, Centre for DNA Fingerprinting and Diagnostics, India
59. Kiyotaka Shiba, Japanese Foundation for Cancer Research, Japan
60. Tetsuo Shibuya, University of Tokyo, Japan
61. Kazuyuki Shimizu, Kyushu Institute of Technology, Japan
62. Worachart Sirawaraporn, Mahidol University, Thailand
63. Daron M Standley Osaka University, Japan
64. Durai Sundar, Indian Institute of Technology Delhi, India
65. Toyotaro Suzumura, Tokyo Institute of Technology, Japan
66. Yoshihiro Taguchi, Chuo University, Japan
67. Takao Takai-Igarashi, Tokyo Medical and Dental University, Japan
68. Yoichi Takenaka, Osaka University, Japan
69. Tin Wee Tan, National University of Singapore, Singapore
70. Hiroshi Tanaka, Tokyo Medical and Dental University, Japan
71. Joo Chuan Tong, Institute for Infocomm Research, A*STAR, Singapore
72. Tetsuro Toyoda, RIKEN BASE (Bioinformatics And Systems Engineering), Japan
73. Tatsuhiko Tsunoda, RIKEN Center for Genomic Medicine, Japan
74. Hiroki R Ueda, RIKEN Center for Developmental Biology, Japan
75. Chandra Verma, Bioinformatics Institute, A*STAR, Singapore
76. Hiroshi Wako, Waseda University, Japan
77. Dennis Paul Wall, CBMI, Harvard University, USA
78. Limsoon Wong, National University of Singapore, Singapore
79. Ueng-Chang Yang, National Yang-Ming University, Taiwan
80. Masayuki Yamamura, Tokyo Institute of Technology, Japan
81. Tetsuya Yomo, Osaka University, Japan
82. Kei Yura, Ochanomizu University, Japan
83. Guanglan Zhang, Dana-Farber Cancer Institute, Harvard University, USA

InCoB2010 Reviewers

We wish thank the following sub-reviewers for contributing with their expertise to the review process:

Shandar Ahmad, Matthew Ardito, Masanori Arita, Daniel Berrar, David Blair, Raffaele Calogero, Qiang Chen, Jose C. Clemente, Adrian Cootes, Jike Cui, Todd DeLuca, Irini Doytchinova, Takaho Endo, Martin Frith, Andre Fujita, Seiya Imoto, Takashi Ishida, Todd Johnson, Jae-Yoon Jung, Takeya Kasukawa, Shuichi Kawano, Asif M. Khan, Pouya Khankhanian, Ryotaro Koike, Tomokazu Konishi, Hidetoshi Kono, William Martin, Hideaki Mizuno, Somay Y. Murayama, Shivashankar Nagaraj, So Nakagawa, Hiroshi Nakashima, Morten Nielsen, Itoshi Nikaido, Hideki Noguchi, Thomas Nordahl Peterson, Takeshi Obayashi, Kohji Okamura, Noriaki Okimoto, Andrew Orr, Sung-Joon Park, Bent Petersen, Jaume Perez-Sanchez, Mark A. Ragan, Kengo Sato, Kenji Satou, Jun Sese, Masafumi Shionyu, Matsuyuki Shirota, Mikita Suyama, Yoshio Tateno, Hiroyuki Toh, Hachiya Tsuyoshi, Lawrence Wee, Chao Xie, and Tetsushi Yada.
