# Supplementary material for: Challenges of the next decade for the Asia Pacific region: 2010 International Conference in Bioinformatics (InCoB 2010)
Source: BMC Genomics. 2010 Dec 2;11(Suppl 4):S1. doi: 10.1186/1471-2164-11-S4-S1 (PMC3005919; doi:10.1186/1471-2164-11-S4-S1)
Supplement: Additional File 2 — InCoB2010 sponsors [file 1471-2164-11-S4-S1-S2.pdf]

Challenges of the next decade for the Asia Pacific region: 2010 International Conference in Bioinformatics (InCoB 2010)
Shoba Ranganathan, Christian Schönbach, Kenta Nakai and Tin Wee Tan

Additional File 2: InCoB2010 Sponsors

Platinum Sponsors

1. Protein Databank Japan(PDBj)
2. Biology Research Center (CBRC), National Institute of Advanced Industrial Science and Technology, Japan (AIST)

Gold Sponsor

1. GenoCon (RIKEN BASE (Bioinformatics And Systems Engineering division)

Silver Sponsors

1. Illumina®
2. MITSUI KNOWLEDGE INDUSTRY CO., LTD.
3. MITSUBISHI SPACE SOFTWARE CO., LTD.
4. Logo Sponsors
5. IMGT®, the international ImMunoGeneTics information system®
6. Hitachi, Ltd.
7. Material Sponsors
8. BioMed Central Ltd.
9. Kyushu Institute of Technology.
